# Osteoprotegerin SNP associations with coronary artery disease and ischemic stroke risk: a meta-analysis

**DOI:** 10.1042/BSR20202156

**Published:** 2020-10-05

**Authors:** Jine Wu, Xiyang Li, Fan Gao, Shanshan Gao, Jun Lyu, Hua Qiang

**Affiliations:** 1 Department of Cardiovascular Medicine, First Affiliated Hospital of Xi’an Jiaotong University, Xi’an, China; 2 Clinical Research Center, First Affiliated Hospital of Xi’an Jiaotong University, Xi’an, China

**Keywords:** Coronary artery disease, Ischemic Stroke, Meta-analysis, Osteoprotegerin, SNP

## Abstract

Osteoprotegerin (OPG) is involved in the development of atherosclerosis and cardio-cerebrovascular disease. The goal of this meta-analysis was to evaluate the association of OPG single nucleotide polymorphisms (SNPs) with coronary artery disease (CAD) and ischemic stroke. A total of 15 eligible studies were extracted from electronic databases. Odds ratios (ORs) were presented, with 95% confidence intervals (CIs), to assess the associations. Meta-analysis was conducted using MetaGenyo, STATA, and Comprehensive Meta-Analysis. Meta-analysis of our data showed that the OPG SNP T950C was significantly associated with increased CAD risk among Asians via recessive (OR 1.55, 95% CI 1.18–2.04, *P*=0.002), CC vs TT (OR 1.57, 95% CI 1.16–2.11, *P*=0.003) and allelic (OR 1.21, 95% CI 1.05–1.38, *P*=0.007) models. No strong associations were observed for the OPG SNP G1181C, T245G and G209A with CAD risk. When evaluating the OPG SNP T245G and T950C associations with ischemic stroke, we found the OPG SNP T245G to be significantly associated with increased risk of ischemic stroke among Chinese via recessive (OR 1.53, 95% CI 1.02–2.29, *P*=0.039) and CC vs AA (OR 1.61, 95% CI 1.07–2.42, *P*=0.021) models. Our results suggested that the OPG SNP T950C was associated with increased risk of CAD among Asians, and the OPG SNP T245G was associated with enhanced ischemic stroke risk among Chinese.

## Introduction

Osteoprotegerin (OPG) is a member of the tumor necrosis factor (TNF) receptor superfamily, also termed as TNF receptor superfamily member 11B (TNFRSF11B) [1]. OPG acts as a decoy receptor, binding to receptor activator of nuclear factor κ-B ligand (RANKL) and blocking its interaction with RANK. Multiple tissues and cells have been described as producing OPG, including bone, heart, endothelial cells, and smooth muscle cells [2]. Although OPG was originally identified as a molecular regulator of bone metabolism, many studies have shown evidence of its involvement in atherosclerosis development. OPG protein and mRNA levels were elevated in human atherosclerotic plaques [3–5]. Additionally, high circulating OPG levels were positively correlated with atherosclerosis progression and the presence of coronary artery disease (CAD) and stroke [6–12]. Increased levels of OPG were also strongly predictive of long-term mortality of CAD patients [13,14]. Based on these observations, OPG was thought to be a potential new biomarker for cardio-cerebrovascular disease. In humans, there are several common single nucleotide polymorphisms (SNPs) identified in the OPG gene, including T950C (T→C, promoter), T245G (T→G, promoter), G209A (G→A, promoter), and G1181C (G→C, exon 1). These SNPs affect circulating OPG levels or protein function [15]. For example, the T950C SNP is significantly associated with increased serum OPG levels, whereas the nonsynonymous polymorphism G1181C can affect cellular secretion of OPG [15]. Numerous studies have assessed their association with CAD and ischemic stroke, but inconclusive results were obtained. To overcome the small sample size problem, we performed an up-to-date meta-analysis to investigate the association between these OPG SNPs and the risk for CAD and ischemic stroke.

## Methods

### Search strategy

A systematic literature search was performed in March 2020 using the following databases: PubMed, Embase, China Science and Technology Journal Database (http://qikan.cqvip.com), and China National Knowledge Infrastructure (http://www.cnki.net). The search terms used in literature search were limited to the following: ‘osteoprotegerin or osteoclastogenesis inhibitory factor or tumour necrosis factor receptor superfamily member 11B’ and ‘coronary heart disease or coronary artery disease or ischemic stroke’ and ‘polymorphism or gene or risk’. We did not apply any geographical restrictions or time restrictions. Reference lists of included studies and of previous related reviews were searched for additional titles. Unpublished data were not considered in this meta-analysis.

### Eligibility criteria

Studies were eligible for inclusion upon meeting the following criteria: (1) case–control studies or cohort studies or retrospective studies; (2) studies evaluated the association of OPG SNPs with CAD or ischemic stroke risk; and (3) studies contained applicable data on allele or genotype distribution in both cases and controls. Exclusion criteria were as follows: (1) familial-based studies or studies using siblings; (2) studies that recruited only cases; (3) published abstracts; and (4) allele or genotype distribution can not be extracted. When studies used similar sources of data, only the study with the largest sample size, or with the most detailed information was selected.

### Data extraction

A standardized data-extraction form was developed for use in our meta-analysis to extract key information from the included articles. Specifically, one reviewer (J.W.) extracted data from the included articles and a second independent reviewer (X.L.) validated the data. The following information was collected: the last name of the first author, country, year of publication, ethnicity, the number of cases and controls, diagnosis method, the mean/range of participants’ age, percentage of male subjects, methods for genotyping OPG SNP, genotype frequency, and Hardy–Weinberg equilibrium (HWE). We did not email the corresponding authors of the eligible studies for additional information.

### Quality assessment

Following the selection of final studies, the Newcastle–Ottawa Scale (NOS) was used to assess study quality [16]. It is widely accepted that the NOS is a reliable quality assessment tool for observational studies. The included studies were evaluated on eight items across three key areas: selection of the participants, comparability of the participants and outcomes. NOS scores of 1–3, 4–6, 7–9 indicated low, intermediate, and high quality, respectively.

### Statistical analysis

The odds ratios (ORs) and 95% confidence intervals (CIs) were used to compare distributions of alleles and genotype contrasts between cases and controls. Allelic, dominant, recessive and co-dominant models were applied to assess the association. Pooled ORs and 95% CIs were calculated using the Mantel–Haenszel fixed-effects model and the DerSimonian–Laird random-effects model [17,18]. Forest plots were generated to visually show the individual study ORs and pooled ORs. Heterogeneity across studies was assessed using the *I^2^* statistic and interpreted based on the study by Higgins et al. [19], where 25, 50, and 75% represented low, moderate, and high heterogeneity, respectively. We performed subgroup analyses according to ethnicity. Sensitivity analysis was conducted to assess the stability of the results. Finally, publication bias was evaluated using funnel plots and Begg’s test. All analyses were performed using Stata, MetaGenyo [20], and Comprehensive Meta-Analysis version 2.

## Results

### Study characteristics

**Figure 1:**
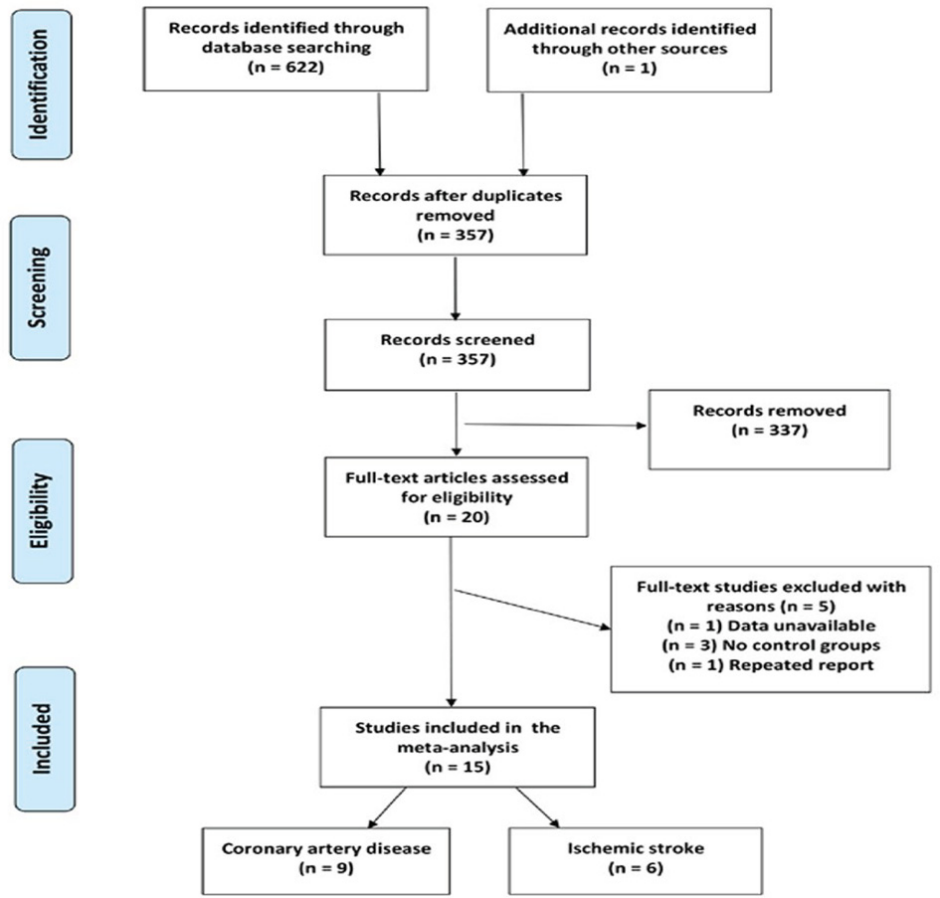
Flow diagram for included studies

The initial electronic database search identified 622 citations. The reference lists of the review articles were hand-searched; one related article was found. Figure 1 shows the identification, screening, and eligibility selection process. After removing duplicates, we evaluated article title and abstracts for the remaining 357 articles and selected 20 articles for full-text review. Following the full-text review, a total of 15 studies were included in the meta-analysis [21–35]. The countries in which the studies had been conducted include China, Japan, Poland, Italy, and Germany. The number of studies with respect to the relationship between the OPG SNPs and CAD risk was 9 [21–28,35]. Six studies provided genotype data for ischemic stroke [29–34]. Descriptive summaries of study characteristics are shown in Tables 1 and 2.

**Table 1:** Characteristics of included studies that evaluated OPG polymorphisms and CAD risk in the meta-analysis

First author	Year	Country	Ethnic Group	CAD diagnosis method	Sample size		Male subjects%		Controls’ MAF	In HWE	NOS	Genotyping method
					Cases	Controls	Cases	Controls				
**T950C**												
Soufi	2004	Germany	Caucasian (German)	Coronary angiography	361	107	100	100	44.9%	Yes	8	DNA sequencing
Ohmori	2006	Japan	Asian (Japanese)	Coronary angiography	405	126	NR	NR	33.3%	Yes	8	PCR-RFLP
Xu	2009	China	Asian (Chinese)	NR	48	102	NR	NR	37.7%	Yes	7	PCR-RFLP
Fang	2010	China	Asian (Chinese)	Coronary angiography	150	150	70.7	63.3	45.0%	Yes	8	PCR-RFLP
Guo	2013	China	Asian (Chinese)	NR	178	312	64.0	62.5	41.0%	Yes	7	PCR-RFLP
Zhao	2019	China	Asian (Chinese)	Coronary angiography	302	226	61.6	45.1	36.9%	Yes	8	PCR-RFLP
**G1181C**												
Soufi	2004	Germany	Caucasian (German)	Coronary angiography	361	107	100	100	27.6%	No	8	DNA sequencing
Fang	2010	China	Asian (Chinese)	Coronary angiography	150	150	70.7	63.3	27.0%	No	8	PCR-RFLP
Celczyńska Bajew	2011	Poland	Caucasian (Poles)	Elective coronary arteriography	31	30	0	0	56.7%	Yes	8	PCR
Hong	2012	China	Asian (Chinese)	Coronary angiography	222	146	62.5	59.6	27.1%	Yes	7	PCR
Luo	2012	China	Asian (Chinese)	Coronary angiography	184	68	NR	NR	18.3%	Yes	6	DNA sequencing
Guo	2013	China	Asian (Chinese)	NR	178	312	64.0	62.5	33.2%	Yes	7	PCR-RFLP
**T245G**												
Soufi	2004	Germany	Caucasian (German)	Coronary angiography	361	107	100	100	6.0%	Yes	8	DNA sequencing
Celczyńska Bajew	2011	Poland	Caucasian (Poles)	Elective coronary arteriography	31	30	0	0	8.0%	Yes	8	PCR
Luo	2012	China	Asian (Chinese)	Coronary angiography	184	68	NR	NR	11.2%	Yes	6	DNA sequencing
Guo	2013	China	Asian (Chinese)	NR	178	312	64.0	62.5	11.5%	Yes	7	PCR-RFLP
**G209A**												
Soufi	2004	Germany	Caucasian (German)	Coronary angiography	361	107	100	100	6.0%	Yes	8	DNA sequencing
Celczyńska Bajew	2011	Poland	Caucasian (Poles)	Elective coronary arteriography	31	30	0	0	8.0%	Yes	8	PCR
Luo	2012	China	Asian (Chinese)	Coronary angiography	184	68	NR	NR	13.2%	Yes	6	DNA sequencing

Abbreviations: MAF, minor allele frequency; NR, not reported; PCR-RFLP, polymerase chain reaction-restriction fragment length polymorphism.

**Table 2:** Characteristics of included studies that evaluated OPG polymorphisms and ischemic stroke risk in the meta-analysis

First author	Year	Country	Ethnic Group	Diagnosis of ischemic stroke	Sample size		Male subjects%		Controls’ MAF	In HWE	NOS	Genotyping method
					Cases	Controls	Cases	Controls				
**T245G**												
Sun	2016	China	Asian (Chinese)	CT or MRI scan	372	165	62.2	43.3	13.6%	Yes	8	PCR-RFLP
Biscetti	2016	Italy	Caucasian (Italian)	CT or MRI scan	487	543	49.7	51.0	33.6%	No	7	PCR-RFLP
Xiong	2018	China	Asian (Chinese)	CT or MRI scan	2835	2224	56.4	65.8	9.2%	Yes	8	High-resolution melt method
Wang	2018	China	Asian (Chinese)	CT or MRI scan	1010	1121	74.1	63.1	12.2%	Yes	8	SNPscan
Fan	2018	China	Asian (Chinese)	CT or MRI scan	213	197	65.7	56.9	13.7%	Yes	7	PCR
**T950C**												
Sun	2016	China	Asian (Chinese)	CT or MRI scan	372	165	62.2	43.3	44.5%	Yes	8	PCR-RFLP
Biscetti	2016	Italy	Caucasian (Italian)	CT or MRI scan	487	548	49.7	51.0	34.2%	Yes	7	PCR-RFLP
Wang	2018	China	Asian (Chinese)	CT or MRI scan	1010	1121	74.1	63.1	40.8%	Yes	8	SNPscan
Fan	2018	China	Asian (Chinese)	CT or MRI scan	213	197	65.7	56.9	40.9%	Yes	7	PCR

Abbreviations: MAF, minor allele frequency; PCR-RFLP, polymerase chain reaction-restriction fragment length polymorphism.

### OPG SNPs and CAD

**Figure 2:**
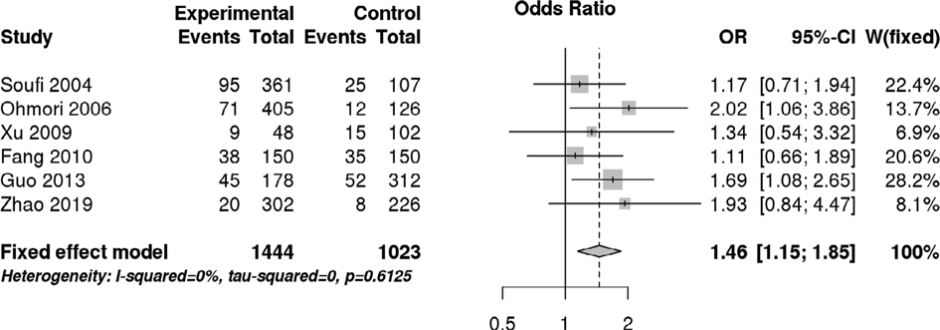
Forest plot demonstrating the association between the OPG SNP T950C and CAD risk via recessive model

**Figure 3:**
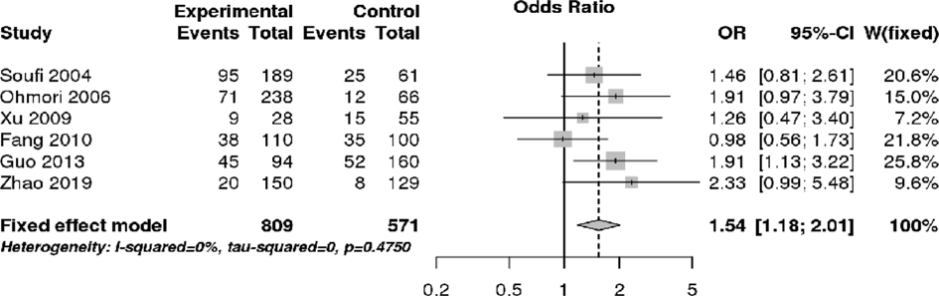
Forest plot demonstrating the association between the OPG SNP T950C and CAD risk via CC vs TT model

Focusing on the OPG SNP T950C, a total of six studies including 1444 patients with CAD and 1023 control subjects were quantitatively analyzed (Table 1). The overall meta-analysis indicated that the OPG SNP T950C was associated with increased CAD risk in recessive (OR 1.46, 95% CI 1.15–1.85, *P*=0.002), CC vs TT (OR 1.54, 95% CI 1.18–2.01, *P*=0.001), and allelic (OR 1.21, 95% CI 1.07–1.39, *P*=0.002) models (Figures 2 and 3, and Table 3). Further subgroup analysis according to ethnicity revealed that the OPG SNP T950C was associated with increased CAD risk among Asians (OR 1.55, 95% CI 1.18–2.04, recessive model, *P*=0.002; OR 1.57, 95% CI 1.16–2.11, CC vs TT, *P*=0.003; OR 1.21, 95% CI 1.05–1.38, allelic model, *P*=0.007) (Table 3).

**Table 3:** Summary of comparative study outcomes for CAD and OPG polymorphisms

OPG SNP	Population	Number of studies	Dominant model			Recessive model			CC vs TT model			Allelic model		
			OR (95% CI)	*P*	*I^2^*	OR (95% CI)	*P*	*I^2^*	OR (95% CI)	*P*	*I^2^*	OR (95% CI)	*P*	*I^2^*
**T950C**	Overall	6	1.45 (0.94–2.24)	0.092	82.6	1.46 (1.15–1.85)	0.002	0	1.54 (1.18–2.01)	0.001	0	1.21 (1.07–1.39)	0.002	0
	Asian	5	1.45 (0.86–2.45)	0.166	86.1	1.55 (1.18–2.04)	0.002	0	1.57 (1.16–2.11)	0.003	0	1.21 (1.05–1.38)	0.007	0
**G1181C**	Overall	6	1.23 (1.02–1.50)	0.034	7.9	1.20 (0.89–1.62)	0.239	49.4	1.35 (0.99–1.84)	0.057	34.3	1.22 (1.05–1.41)	0.009	37.5
	Asian	4	1.21 (0.98–1.51)	0.082	11.7	1.11 (0.77–1.60)	0.566	62.4	1.38 (0.96–1.98)	0.085	60.9	1.24 (0.94–1.65)	0.130	61.4
	Caucasian	2	1.30 (0.86–1.97)	0.208	48.2	1.40 (0.82–2.39)	0.212	38.9	1.29 (0.72–2.31)	0.396	0	1.29 (0.95–1.76)	0.100	0
	Follow HWE	4	1.12 (0.88-1.43)	0.367	21.3	1.45 (0.99–1.78)	0.060	70.5	1.33 (0.88–2.01)	0.176	59.8	1.17 (0.97–1.40)	0.105	59.0
**T245G**	Overall	4	0.98 (0.71–0.134)	0.879	0	1.50 (0.52–4.29)	0.454	6.3	1.47 (0.51–4.24)	0.474	3.5	1.02 (0.76–1.36)	0.919	0
	Asian	2	0.98 (0.68–1.43)	0.924	0	1.50 (0.52–4.29)	0.454	6.3	1.47 (0.51–4.24)	0.474	3.5	1.03 (0.74–1.45)	0.847	0
	Caucasian	2	0.96 (0.53–1.74)	0.893	0	NA	NA	NA	NA	NA	NA	0.96 (0.54–1.71)	0.897	0
**G209A**	Overall	3	0.93 (0.60–1.43)	0.740	0	4.99 (0.28–89.76)	0.276	NA	4.64 (0.26–83.84)	0.299	NA	1.01 (0.67–1.51)	0.978	0
	Caucasian	2	1.01 (0.56–1.82)	0.987	0	NA	NA	NA	NA	NA	NA	1.01 (0.57–1.78)	0.988	0

Abbreviation: NA, not applicable.

Six studies involving 1126 CAD patients and 813 controls provided results on the association of the OPG SNP G1181C with CAD risk (Table 1). The pooled analyses showed that the SNP G1181C was associated with increased CAD risk in dominant and allelic models, but not in recessive and CC vs GG models (Table 3). However, when removing two studies that were not in line with HWE [21,24], we found no association between this SNP and CAD risk (OR 1.12, 95% CI 0.88–1.43, dominant model, *P*=0.367; OR 1.45, 95% CI 0.99–1.78, recessive model, *P*=0.060; OR 1.33, 95% CI 0.88–2.01, CC vs GG model, *P*=0.176; OR 1.17, 95% CI 0.97–1.40, allelic model, *P*=0.105) (Table 3). Stratification analysis based on ethnicity did not find a significant association between CAD risk and the OPG SNP G1181C in Asians or Caucasians (Table 3).

Four studies including 754 cases and 517 controls examined the associations of the SNP T245G with CAD risk, while three studies with 576 cases and 205 control subjects performed evaluation of the association between the OPG SNP G209A and CAD (Table 1). In the overall meta-analysis and stratification analysis based on ethnicity, we found no association between these SNPs and CAD risk (Table 3).

### OPG SNPs and ischemic stroke

**Figure 4:**
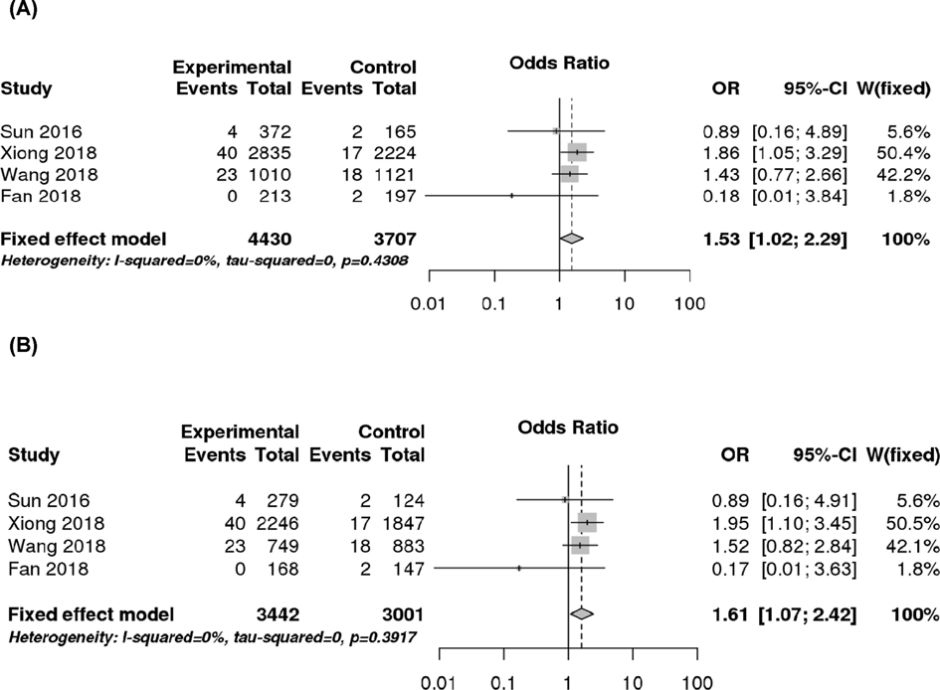
Forest plots demonstrating the association between the OPG SNP T245G and ischemic stroke risk among Chinese (**A**) Recessive model; (**B**) CC vs AA model.

The OPG SNP T245G associations with ischemic stroke risk were evaluated in five studies involving 4917 cases and 4250 controls, whereas four studies including 2082 cases and 2031 controls focused on the T950C polymorphism (Table 2). The SNP T245G was significantly associated with ischemic stroke in recessive (OR 1.53, 95% CI 1.02–2.29, *P*=0.039) and CC vs AA (OR 1.61, 95% CI 1.07–2.42, *P*=0.021) models (Figure 4 and Table 4) among Chinese subjects. No significant association was found between the OPG SNP T950C and ischemic stroke when combining all studies together or performing subgroup analysis for Chinese (Table 4). Subgroup analysis could not be performed for Caucasians, as there was only one small Caucasian study.

**Table 4:** Summary of comparative study outcomes for ischemic stroke and OPG polymorphisms

OPG SNP	Population	Number of studies	Dominant model			Recessive model			CC vs AA model			Allelic model		
			OR (95% CI)	*P*	*I^2^*	OR (95% CI)	*P*	*I^2^*	OR (95% CI)	*P*	*I^2^*	OR (95% CI)	*P*	*I^2^*
**T245G**	Overall	5	1.32 (0.97–1.79)	0.08	86.1	1.85 (0.86–3.96)	0.115	79.7	1.93 (0.78–4.81)	0.156	85.1	1.29 (0.93–1.80)	0.132	91.9
	Chinese	4	1.18 (0.97–1.43)	0.106	56.7	1.53 (1.02–2.29)	0.039	0	1.61 (1.07–2.42)	0.021	0	1.15 (0.95–1.39)	0.148	62.0
	Follow HWE	4	1.18 (0.97–1.43)	0.106	56.7	1.53 (1.02–2.29)	0.039	0	1.61 (1.07–2.42)	0.021	0	1.15 (0.95–1.39)	0.148	62.0
**T950C**	Overall	4	1.17 (0.79–1.74)	0.431	86.5	1.21 (0.57–2.59)	0.621	94.1	1.29 (0.54–3.11)	0.566	94.6	1.14 (0.76–1.71)	0.534	0.943
	Chinese	3	0.97 (0.83–1.12)	0.906	0	0.91 (0.75–1.10)	0.324	29.8	0.91 (0.73–1.12)	0.379	0	0.96 (0.86–1.06)	0.415	0

Abbreviation: NA, not applicable.

### Heterogeneity and publication bias

When evaluating the association between OPG polymorphisms and CAD, there was no significant heterogeneity (*I^2^* < 50%) in most pooled estimates (Table 3). Significant heterogeneity was found between studies evaluating OPG polymorphisms and ischemic stroke (Table 4). However, when subgroup analysis was performed, heterogeneity was greatly reduced in the four genetic models for the SNP T950C and in recessive and CC vs AA models for the SNP T245G (Table 4). The number of included study for each SNP was less than ten, precluding us from creating funnel plots. So we used the Begg’s test to evaluate publication bias. The data did not suggest the presence of publication bias (*P*>0.10). Sensitivity analysis was performed to evaluate the robustness of the observed outcomes. The relevant pooled ORs were not considerably altered after excluding any study (Supplementary Information).

## Discussion

CAD is the major cause of fatality and disability for both men and women in the world. It accounts for more than 7 million deaths each year. Due to its high incidence, prevalence, and mortality, the identification of a specific biomarker that can be used to estimate an individual person’s risk of developing CAD would be invaluable. OPG is a cytokine belonging to the TNF receptor superfamily. Initial studies found that OPG was an important mediator of bone remodeling, but recent experimental and observational studies demonstrated that OPG may also contribute to the development of atherosclerosis and CAD. In atherosclerotic plaques, elevated OPG mRNA and protein levels were observed [3–5]. Several observational studies found that serum levels of OPG were independently correlated with the presence and severity of CAD after adjusting for traditional risk factors such as hypertension and smoking in logistic regression models [8,10,11]. Moreover, increased OPG levels strongly predicted adverse clinical outcomes in patients with CAD. These findings raised the possibility that genetic polymorphisms in the OPG might affect an individual person’s risk of CAD.

We evaluated the association between CAD and several OPG SNPs, including T950C, G1181C, T245G, and G209A in the present study. These are common SNPs within the OPG gene and potentially affect the OPG protein’s structural or functional properties. Through a comprehensive meta-analysis using nine studies, we found that the OPG SNP T950C was associated with increased risk of CAD (OR 1.46 for recessive model; OR 1.54 for CC vs TT model; OR 1.21 for allelic model), especially in Asian subjects. Studies of the OPG SNP G1181C, T245G, and G209A showed no significant association with CAD risk.

Jia et al. [36] meta-analyzed the association of the OPG SNP T950C, G1181C, T245G, and G209A with CAD risk, including two Chinese-language and four English-language studies in 2017. Their results showed an association of the OPG SNP T950C with CAD risk, in line with our findings. The sample size of their meta-analysis was not large; only three studies were pooled to estimate the association. In our study, we increased the sample size by including three new studies published since the previous meta-analysis and confirmed that the OPG SNP T950C was an important genetic risk factor for CAD. Although Jia et al. also reported an association between the OPG SNP G1181C and CAD risk, this association was not confirmed by our meta-analysis [36]. We did not detect any significant effect of the SNP G1181C on CAD among either Asians or Caucasians. Additionally, sensitivity analysis by excluding the studies that did not follow HWE showed no association. With respect to the OPG SNP G209A and T245G, the results obtained by Jia et al. [36] were in agreement with the present meta-analysis, indicating no effect of them on CAD risk.

The OPG SNP T950C lies in the OPG gene promoter (129 bp upstream from the TATA box). Previous studies reported that the SNP was significantly and independently associated with increased circulating OPG levels [37]. It was also shown to affect atherosclerotic plaque stability [38,39]. The role of the SNP T950C in atherosclerosis development and plaque stability may help explain its association with increased CAD risk, but further functional assays are needed to investigate the precise mechanisms linking the SNP T950C and CAD. Moreover, the SNP T950C may be linked to other functional polymorphisms in the RANKL/RANK/OPG signaling pathway. A recent meta-analysis of GWAS identified new significant loci on chromosome 17q11.2 as well as chromosome 14q21.2 that associated with circulating OPG levels [40]. Future research is required to explore potential gene–gene interactions between OPG SNPs and other functional variants that affect circulating OPG levels.

Ischemic stroke is the most common form of stroke. Previous studies have observed a relation between a higher level of OPG and ischemic stroke severity and outcome [6,9,12]. To our knowledge, no published meta-analyses in the literature have evaluated the effect of OPG polymorphisms on ischemic stroke risk. Therefore, we meta-analyzed the OPG SNP T245G and T950C and their relationships with ischemic stroke in the present study. The analysis showed the OPG SNP T245G to be associated with increased risk of ischemic stroke among Chinese via recessive and CC vs AA models. The SNP T245G located in the promoter region is functional for the OPG gene. It has been shown to affect OPG expression level and might be involved in atherosclerotic lesion progression [32]. Wang et al. [41] showed that the SNP T245G was correlated with a worse outcome in patients with large artery atherosclerosis stroke. Our meta-analysis did not evaluate the OPG SNP effects among Caucasians, due to limited published studies in the literature. However, it is noteworthy that one small Caucasian study performed in an Italian population found significant effects of the SNP T245G and T950C in ischemic stroke risk. This interesting finding needed to be confirmed in other Caucasian populations.

Our meta-analysis has some limitations. First, for some studies, the selection of CAD patients might bias the results. These studies recruited hospital patients undergoing diagnostic coronary angiography for suspected CAD, who were generally a highly selected population [21,22,26]. Second, the relation between the SNP T245G and T950C and ischemic stroke was only evaluated among Italian and Chinese. It needs to be further assessed in more ethnic groups. Third, heterogeneity was not fully eliminated by subgroup analysis when evaluating the relation between the SNP T245G and ischemic stroke. Heterogeneity could also be related to other factors such as study design and patients’ characteristics. Fourth, the pooled ORs were not adjusted for patient characteristics including body mass index, arterial hypertension, and hypercholesterolemia, due to a small size or insufficient information. Finally, subgroup analysis by subtype of CAD or ischemic stroke was unavailable.

In summary, the present meta-analytic study identified a significant association between the OPG SNP T950C and the risk of CAD among Asians via recessive, CC vs TT and allelic models. Additionally, we showed the OPG SNP T245G to be significantly associated with increased ischemic stroke risk among Chinese via recessive and CC vs AA models. We are hopeful that our findings would be further evaluated by adequately powered and well-designed studies, especially in non-Asian populations.

## Abbreviations

CAD: coronary artery disease
CI: confidence interval
GWAS: genome-wide association study
HWE: Hardy–Weinberg equilibrium
NOS: Newcastle–Ottawa Scale
OPG: osteoprotegerin
OR: odds ratio
RANKL: receptor activator of nuclear factor κ-B ligand
SNP: single nucleotide polymorphism
TNF: tumor necrosis factor
